# Baseline serum MMP-3 levels in patients with Rheumatoid Arthritis are still independently predictive of radiographic progression in a longitudinal observational cohort at 8 years follow up

**DOI:** 10.1186/ar3734

**Published:** 2012-02-07

**Authors:** Mark Houseman, Catherine Potter, Nicola Marshall, Rachel Lakey, Tim Cawston, Ian Griffiths, Steven Young-Min, John D Isaacs

**Affiliations:** 1 Institute of Cellular Medicine, Musculoskeletal Research Group, Newcastle University, 4th Floor Catherine Cookson Building, The Medical School, Framlington Place, Newcastle upon Tyne, NE2 4HH, UK; 2 Musculoskeletal Services, Freeman Hospital, Freeman Road, High Heaton, Newcastle upon Tyne, NE7 7DN, UK; 3 Rheumatology, Queen Alexandra Hospital, Southwick Hill Road, Portsmouth, PO6 3LY, UK

## Abstract

**Introduction:**

At present, there is no reliable tool for predicting disease outcome in patients with rheumatoid arthritis (RA). We previously demonstrated an association between specific baseline biomarkers/clinical measures including matrix metalloproteinase-3 (MMP-3) and 2-year radiographic progression in patients with RA. This study further evaluates the predictive capability of these baseline variables with outcome extended over 8-years.

**Methods:**

Fifty-eight of the original cohort (*n *= 118) had radiographic progression from baseline to mean 8.2-years determined using the van der Heijde modified Sharp method. The contribution of each predictor variable towards radiographic progression was assessed with univariate and multivariate analyses.

**Results:**

Traditional factors (including erythrocyte sedimentation rate, C-reactive protein, anti-cyclic citrullinated peptide (anti-CCP), and rheumatoid factor) and biomarkers of tissue destruction (including MMP-3, C-telopeptide of type II collagen, cartilage oligomeric matrix protein, and tissue inhibitor of metalloproteinase 1) measured at baseline were associated with radiographic progression at endpoint. Multivariate logistic regression identified anti-CCP seropositivity [OR 9.29, 95%CI: 2.29-37.64], baseline elevated MMP-3 [OR 8.25, 95%CI: 2.54-26.78] and baseline radiographic damage [OR 5.83, 95%CI: 1.88-18.10] as the strongest independent predictors of radiographic progression. A model incorporating these variables had a predictive accuracy of 0.87, assessed using the area under the receiver operating characteristic curve.

**Conclusion:**

In our cohort with onset of RA symptoms < 2-years, multivariate analysis identified anti-CCP status and baseline MMP-3 as the strongest independent predictors of radiographic disease outcome at 8.2-years. This finding suggests determination of baseline MMP-3, in conjunction with traditional serologic markers, may provide additional prognostic information for patients with RA. Furthermore, these findings highlight the importance of continued research into a broad range of biomarkers as potential predictors of joint damage.

## Introduction

Current paradigms for management of patients with rheumatoid arthritis (RA) dictate early aggressive therapy in treatment-to-target strategies, aiming for remission of symptoms [1]. In turn this prevents joint destruction and associated co-morbidities, including cardiovascular complications. A particular concern regarding these principles, however, is that some patients with RA may remit with less aggressive treatment regimes, exposing a proportion of patients to unnecessary medications and their associated risks. Ideally it should be possible to study baseline clinical characteristics and laboratory biomarkers of RA patients and prescribe according to a predictive algorithm, so-called personalised medicine. Current algorithms, however, while predictive at a population level, have insufficient power to guide treatment of the individual patient [2].

Some baseline clinical and demographic markers (e.g. female sex, older age, rheumatoid factor (RF), anti-cyclic citrullinated peptide (anti-CCP) seropositivity, raised C-reactive protein (CRP) or erythrocyte sedimentation rate (ESR)) have been associated with a poor prognosis [3]. Surprisingly, none of these markers specifically reflect ongoing destructive processes within bone and synovium. Utilising observations from our own early arthritis cohort, we previously reported a multivariate logistic regression of various biomarkers at baseline. Our data suggested that a model consisting of matrix metalloproteinase-3 (MMP-3) and C-telopeptide of type II collagen (CTX-II) performed better than ESR and CRP in predicting two-year radiographic progression [4]. To examine the robustness of our model over time we now report follow-up data over eight years, additionally incorporating anti-CCP status. We demonstrate that the measurement of serum MMP-3 levels at baseline enhances the predictive value of anti-CCP in determining long-term radiographic outcome in patients with RA. Hence, these findings suggest that assessment of baseline MMP-3 and other biomarkers of joint destruction, in conjunction with existing serological markers and clinical measures, may provide additional long-term prognostic information for patients with RA.

## Materials and methods

### Patients

The original cohort (*n *= 118, RA symptoms for less than two years) presented between 1998 and 2000. Patient demographics, inclusion criteria and study protocol were described previously [4]. Sixty-two patients were revisited in 2007, at mean follow up of 8.2 years. Four patients were excluded with an alternative subsequent diagnosis (three psoriatic arthritis and one systemic lupus erythematosus). The remainder were either deceased (*n *= 16), lost to follow up (*n *= 16) or declined participation (*n *= 24). Baseline CRP levels were higher in revisited patients but there were no other significant differences in baseline characteristics [see Additional data file 1]. Treatment during the intervening period was decided according to current local practice with sequential disease-modifying anti-rheumatic drug (DMARD) mono or combination therapy. Five patients (evenly split between low (*n *= 2) and high (*n *= 3) progressor groups) subsequently received anti-TNF biologic therapy. At the extension visit serum was taken to determine anti-CCP status (Axis-Shield Diagnostics Limited, Dundee, Scotland, UK). Ethical approval was obtained from Newcastle and North Tyneside Research Ethics Committee and all patients gave informed consent to take part in this extension study.

### Scoring of radiographs and division of cohort

**Figure 1:**
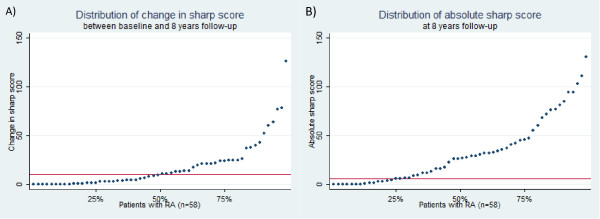
**Cumulative distribution plots for outcome measures**. **(a) **Change in Sharp's score from baseline to 8.2-years follow up. **(b) **Total Sharp's score at 8.2-years follow up. The red line indicates the median and lower quartile cut-off points, respectively. RA, rheumatoid arthritis.

Posteroanterior radiographs of the hands/wrists and feet were collected at baseline and 8.2 years. One observer (MH) was trained to apply the van der Heijde modified Sharp scoring (SHS) method [5]. An intraclass correlation coefficient of 0.91 was obtained assessing the same test set (20 pairs of random radiographs) two weeks apart. Subsequently, radiographs were scored in chronological order but blinded to identity. The baseline SHS was subtracted from the 8.2-year SHS for each patient and a value representing radiographic progression obtained. Individuals were allocated to low- or high-progressive groups based on the median SHS change (Figure 1). Additional analysis was performed using only the total SHS at 8.2 years to determine the 'absolute' radiographic outcome. For this analysis the cohort was sub-divided into non-progressors and progressors using the lower quartile value derived from the total SHS distribution (Figure 1).

### Statistical analysis

A more detailed discussion regarding the use of non-parametric analyses was provided previously [4]. Briefly, association between baseline values of each potential predictor and radiographic progression/absolute radiographic outcome at 8.2 years, considered both as a continuous variable (Spearman's rank correlation) and as a binary variable (Mann Whitney U test/Chi-square test/Student's t-test), was analysed. Univariate and forward stepwise (entry probability = 0.05, removal probability = 0.1) logistic regression were subsequently performed to assess the contribution of each associated marker and to identify the most predictive multivariate model, respectively. For this analysis baseline variables were dichotomised into positive or negative for autoantibodies, or high and low values based on median values obtained in this study. The predictive accuracy of each model was assessed using the area under the receiver operating characteristic curve (ROC). All statistical calculations were performed in Stata^® ^(StataCorp, College Station, Texas, USA).

## Results

### Division of the cohort based on radiographic progression at 8.2 years

Fifty-eight (49%) patients from the original cohort were analysed in the extension study. The median change in SHS of 10.5 determined whether individuals were assigned to the low (*n *= 29, SHS ≤10.5) or high progressor (*n *= 29, > 10.5) group. Table 1 highlights baseline demographic and clinical characteristics across the two groups.

**Table 1:** Comparison of baseline demographic characteristics, clinical and biomarker measures in radiographic progressor groups at 8.2 years

	Low Progressors*	High Progressors*	Comparison		SRC	
Cohort characteristics	(*n *= 29)	(*n *= 29)	z	***P***^**$**^	rho	*P*
**Demographic**						
Female, no. (%)	22 (76%)	15 (52%)	3.66	0.100^**†**^	-	NA
Age, mean ± SD, years	52 ± 10	55 ± 12	0.97	0.334^**‡**^	-	NA
Disease duration, days	260 (169, 412)	242 (146, 384)	-0.59	0.555	-	NA
DAS28	5.1 (4.0, 6.1)	6.2 (4.8, 6.8)	1.73	0.083	-	NA
HAQ	1.3 (0.9, 2.0)	1.5 (1.0, 2.3)	0.90	0.370	-	NA
No. DMARDs^φ^	2 (1, 2)	2 (2, 3)	1.81	0.070	-	NA
MTX, no. (%)^φ^	8 (28)	21 (72)	11.66	0.001^**†**^	-	NA
**Traditional measures**						
Total SHS	3 (0, 8)	16 (6, 28)	4.09	< 0.001	0.51	< 0.001
ESR, mm/hour	19 (11, 39)	34 (20, 50)	2.33	0.020	0.35	0.008
CRP mg/L	8 (5, 20)	34 (15, 65)	3.42	< 0.001	0.46	< 0.001
RF positive, no. (%)	19 (66%)	26 (90%)	4.86	0.056^**†**^	0.47	< 0.001
Anti-CCP +ve, no. (%)^φ^	14 (48%)	26 (90%)	11.60	0.001^**†**^	0.23	0.077
SJC	6 (2, 12)	10 (5, 15)	1.80	0.072	0.19	0.145
TJC	14 (5, 18)	15 (7, 18)	0.47	0.640	0.06	0.675
CGA on VAS, mm	24 (10, 57)	54 (38, 68)	2.48	0.013	0.34	0.010
PGA on VAS, mm	39 (27, 48)	47 (24, 65)	0.53	0.600	0.10	0.447
**Biomarkers**						
MMP-3, ng/mL	51 (32, 86)	137 (86, 359)	3.83	< 0.001	0.46	< 0.001
CTX-II, μg/mmol	0.14 (0.08, 0.25)	0.36 (0.19, 0.57)	3.40	< 0.001	0.40	0.003
COMP, U/L	10 (8, 11)	13 (11, 16)	3.27	0.001	0.39	0.003
TIMP-1, ng/mL	647 (466, 719)	817 (669, 902)	2.34	0.019	0.29	0.029

*Except where indicated otherwise, values are median (25^th ^and 75^th ^percentiles). ^$^Determined by Mann Whitney U test unless indicated otherwise. ^†^Determined by Fisher's two-sided exact test. ^‡^Determined by Student's t test. ^φ ^Value taken at 8.2-year follow-up visit.

Anti-CCP, anti-cyclic citrullinated peptide; CGA, clinician's global assessment; COMP, cartilage oligomeric matrix protein; CRP, C-reactive protein; CTX-II, C-telopeptide of type II collagen; DAS, disease activity score; DMARD, disease-modifying anti-rheumatic drug; ESR, erythrocyte sedimentation rate; HAQ, health assessment questionnaire; MMP-3, matrix metalloproteinase-3; MTX, methotrexate; NA, not applicable; NS, not significant; PGA, patient's global assessment; RF, rheumatoid factor; SD, standard deviation; SHS, van der Heijde modified Sharp score; SJC, swollen joint count; SRC, Spearman's rank correlation; TIMP-1, tissue inhibitor of metalloproteinase 1; TJC, tender joint count; VAS, visual analogue score.

### Predictors of radiographic progression at 8.2 years

Mann Whitney analysis of traditional markers indicated that high radiographic progressors had significantly elevated measures of ESR, CRP, SHS and clinician's global assessment (CGA) at baseline compared with low progressors (Table 1). Extending our previous findings, baseline levels of MMP-3, CTX-II, cartilage oligomeric matrix protein (COMP), and tissue inhibitor of metalloproteinase 1 (TIMP-1) remained significantly higher in high radiographic progressors. It should be noted that there were significant correlations between levels of these various markers [see Additional data file 2]. In addition, baseline RF and anti-CCP status (determined at 8.2 years) were more frequently positive in the highly progressive patients. These findings were reproduced using Spearman's rank correlation analysis assessing radiographic change as a continuous variable.

Biomarkers and clinical measures demonstrating association in the exploratory analyses were dichotomised and further investigated by univariate logistic regression (Table 2). Subsequently, stepwise logistic regression identified anti-CCP positivity, baseline elevated MMP-3 and baseline SHS as the strongest independent predictors of radiographic progression at 8.2 years (chi-squared = 14.97, *P *= 0.002). The predictive accuracy of this model, as assessed by ROC, was superior to either factor alone (combined area under the curve (AUC) = 0.87, anti-CCP AUC = 0.71, MMP-3 AUC = 0.74, and SHS AUC = 0.71). The positive predictive values (PPV) and negative predictive values (NPV) of the combined model were 81% and 85%, respectively.

**Table 2:** Performance of baseline biomarkers as predictors of radiographic progression and absolute radiographic outcome at 8.2 years with univariate analysis

	Low vs high radiographic progression				Absolute radiographic outcome, progressors vs non-progressors			
Baseline characteristics	PPV (%)	NPV (%)	*P*	OR (95% CI)	PPV (%)	NPV (%)	P	OR (95% CI)
**Traditional measures**								
SHS (> 7^┴^)	71.43	70.00	0.002	5.83 (1.88, 18.10)	-	-	-	-
ESR (> 20 mm/hr^∇^)	60.00	65.22	0.064	2.81 (0.94, 8.39)	85.71	39.13	0.036	3.86 (1.09, 13.65)
CRP (> 5 mg/L^∇^)	59.52	75.00	0.024	4.41 (1.22, 16.00)	85.71	50.00	0.007	6.00 (1.62, 22.16)
Anti-CCP* (titer > 6 U/mL^∇^)	65.00	83.33	0.002	9.29 (2.29, 37.64)	90.00	55.56	0.001	11.25 (2.80, 45.16)
RF (titer > 40 U/mL^∇^)	57.78	76.92	0.036	4.56 (1.10, 18.86)	77.78	30.77	0.528	1.56 (0.39, 6.13)
CGA on VAS (> 49.00 mm^┴^)	60.71	60.00	0.118	2.32 (0.81, 6.64)	85.71	33.33	0.098	3.00 (0.82, 11.04)
**Biomarkers of joint damage**								
MMP-3 (> 85.79 ng/mL^┴^)	75.00	73.33	< 0.001	8.25 (2.54, 26.78)	89.29	36.67	0.029	4.82 (1.18, 19.74)
CTX-II (> 0.20 μg/mmol^┴^)	66.67	66.67	0.016	4.00 (1.29, 12.40)	85.19	37.04	0.070	3.38 (0.91, 12.64)
COMP (> 11.20 U/L^┴^)	67.86	68.97	0.007	4.69 (1.54, 14.34)	89.29	37.93	0.024	5.09 (1.24, 20.92)
TIMP-1 (> 688.68 ng/mL^┴^)	65.52	65.52	0.020	3.61 (1.22, 10.66)	86.21	34.48	0.073	3.29 (0.89, 12.12)

* = Anti-CCP taken at 8.2-year follow-up visit. ^┴ ^= indicates derived from median value in this study, ^∇ ^= indicates derived from established normal value

95% CI, 95% confidence interval; Anti-CCP, anti-cyclic citrullinated peptide; CGA, clinician's global assessment; COMP, cartilage oligomeric matrix protein; CRP, C-reactive protein; CTX-II, C-telopeptide of type II collagen; ESR, erythrocyte sedimentation rate; MMP-3, matrix metalloproteinase-3; NPV, negative predictive value; OR, odds ratio; PPV, positive predictive value; RF, rheumatoid factor; SHS, van der Heijde modified Sharp score; TIMP-1, tissue inhibitor of metalloproteinase 1; VAS, visual analogue score.

### Predictors of absolute radiographic outcome at 8.2 years

Subanalyses were undertaken incorporating the patient group deemed to have minimal radiographic progression. This utilised only the total SHS at 8.2 years (median value = 26.5, lower quartile = 6, upper quartile = 45.8). Patients were dichotomised into two groups, termed non-progressors (*n *= 14) and progressors (*n *= 44), based on the lower quartile SHS. Univariate logistic regression indicated traditional measures (ESR, CRP and anti-CCP) and biomarkers (MMP-3 and COMP) were also significant predictors of absolute radiographic outcome (Table 2). Furthermore, stepwise logistic regression identified anti-CCP positivity and baseline elevated MMP-3 as independent predictors of radiographic outcome (chi-squared = 12.40, *P *= 0.002). The predictive accuracy of this model was superior to either factor alone (combined AUC = 0.84, anti-CCP AUC = 0.77, and MMP-3 AUC = 0.68). The PPV and NPV of the combined model were 87% and 64%, respectively.

## Discussion

This extension study was conceived to further evaluate the performance of selected biochemical/serological markers and routine clinical measures in predicting radiographic disease outcome in a well-characterised RA cohort. Apart from the inclusion of anti-CCP status, the main methodological departure from the original study was the use of the van der Heijde modified Sharp radiograph scoring system, which is more sensitive for detecting temporal radiographic change in early RA [6]. The change in SHS from baseline to 8.2 years was utilised to reflect progression due to the underlying disease process [7]. When considering the distribution of this measure (Figure 1), patients were dichotomised into low and high progressors using the median, an approach validated by correlation analyses using change in SHS as a continuous variable. Additionally the absolute radiographic prognosis at 8.2 years was assessed to isolate the patient group that might require less aggressive treatment and thus limit exposure to therapies that carry potentially serious side effect profiles.

Various baseline traditional factors including ESR, CRP and RF, and biomarkers of cartilage and collagen breakdown including CTX-II, COMP and TIMP-1 were associated with radiographic progression at 8.2 years, consistent with other studies [3,4]. Overall, the strongest predictors were anti-CCP (odds ratio (OR) = 9.29, 95% confidence interval (CI) = 2.29 to 37.64) and MMP-3 (OR = 8.25, 95% CI = 2.54 to 26.78), followed by baseline SHS (OR = 5.83, 95% CI = 1.88 to 18.10). Similar findings were observed in the subanalyses investigating predictors of absolute radiographic prognosis. Baseline anti-CCP seropositivity has been associated previously with radiographic progression [8,9]. Notably, the anti-CCP status in our cohort was determined at follow up (8.2 years) rather than baseline as it was not routinely available at the time of primary analysis. However, as other studies have shown positivity is generally stable over time, we have used this as a surrogate of patients' baseline status [10]. The predictive accuracy of our model increased from 71% to 87% (PPV = 81% and NPV = 85%) with the addition of baseline MMP-3 and baseline SHS to anti-CCP, an improvement of potential clinical relevance. The inclusion of baseline radiographic SHS score in our model contrasts with data from the ASPIRE and ATTRACT biologic therapy trials, in which baseline radiographic damage did not predict outcome [11]. However, 82% to 99.1% of the ASPIRE and ATTRACT participants had erosions at entry [11] compared with 14% in our cohort, and it is now well established that TNF blockade is more effective than traditional DMARD therapy at inhibiting radiographic progression. Furthermore, recent data from the BeSt study highlighted seropositivity (anti-CCP or RF), baseline erosion score and CRP as predictors of rapid radiographic progression [12]. Nonetheless, excluding baseline SHS from our model, to provide more accessible prognostic information in the clinic setting, had only a modest effect on its performance (AUC = 0.83, PPV = 89% and NPV = 74%).

In our original study, stepwise logistic regression identified baseline MMP-3 with CTX-II as the strongest predictive model of radiographic outcome at two-years follow up. As for other biomarkers investigated, CTX-II remained a strong predictor of radiographic outcome at 8.2 years following univariate analysis; however, it appeared to be no longer an independent predictor following multivariate analysis. This finding may reflect the combination of reduced patient numbers and the correlation between CTX-II and MMP-3 [see Additional data file 2]. Indeed, it should be noted that several biomarkers performed similarly well in the univariate analysis taking into account the wide confidence intervals. Hence future studies, while attempting to validate and assess the clinical value of our findings, should continue to study a broad range of biomarkers as potential predictors of joint damage. The reduced size of the follow-up cohort is a weakness of the current analysis but, apart from baseline CRP (which was higher in revisited patients), there were no other significant differences in baseline characteristics between those patients who were and were not revisited as part of the current study [see Additional data file 1].

Notwithstanding the above caveats and in keeping with results from the primary analysis, our eight-year data continue to highlight MMP-3 as a strong predictor of radiographic progression. MMPs play a pivotal role in cartilage destruction and although they might provide a means to monitor disease activity, response to treatment and prognosis [13], this has yet to be fully established [14]. Supporting evidence for the prognostic significance of MMP-3 in RA, however, includes its expression in RA synovial tissue and decreasing serum MMP-3 levels following DMARD treatment [4,15]. Baseline MMP-3 levels have previously been shown to correlate with joint destruction in studies with up to three years of observations [4,16-21] but, to our knowledge, ours is the first to report that this predictive effect is maintained at eight years of follow up.

## Conclusions

We have demonstrated that the measurement of serum MMP-3 levels at baseline adds to the predictive value of anti-CCP in determining long-term radiographic outcome in patients with RA. These findings support the notion that MMP-3 influences important pathological processes that are distinct from anti-CCP and are fundamental to the development of radiographic progression. In addition, these findings indicate that assessment of biomarkers that reflect joint destruction, such as baseline MMP-3, in conjunction with existing serological markers and clinical measures, may provide potentially important prognostic information for patients with early RA. We acknowledge the modest size of our cohort but suggest that validation of these promising findings in additional prospective studies is now warranted.

## Abbreviations

Anti-CCP: anti-cyclic citrullinated peptide; AUC: area under the curve; CGA: clinician's global assessment; CI: confidence interval; COMP: cartilage oligomeric matrix protein; CRP: C-reactive protein; CTX-II: C-telopeptide of type II collagen; DMARD: disease-modifying anti-rheumatic drug; ESR: erythrocyte sedimentation rate; MMP-3: matrix metalloproteinase-3; NPV: negative predictive value; OR: odds ratio; PPV: positive predictive value; RA: rheumatoid arthritis; RF: rheumatoid factor; ROC: receiver operating characteristic; SHS: van der Heijde modified Sharp score; TIMP-1: tissue inhibitor of metalloproteinase 1; TNF: tumour necrosis factor.

## Competing interests

The authors declare that they have no competing interests.

## Authors' contributions

MH, SYM, TC, IG and JDI conceptualised and designed the study. MH and NM undertook recruitment of patients and collection of clinical data. MH and RL undertook laboratory analyses. CP, MH and JDI analysed and interpreted the data. MH, CP and SYM drafted the manuscript with contributions from all authors. All authors have read and approved the manuscript for publication.

## Supplementary Material

Additional file 1 **Baseline characteristics across participating and non-participating subjects**. Table presenting comparison analyses of baseline characteristics across participating and non-participating subjects. Click here for file

Additional file 2 **Correlation between baseline biomarker measures**. Table presenting degree of correlation between novel biomarker measures. Click here for file
