# Supplementary material for: Baseline serum MMP-3 levels in patients with Rheumatoid Arthritis are still independently predictive of radiographic progression in a longitudinal observational cohort at 8 years follow up
Source: Arthritis Res Ther. 2012 Feb 7;14(1):R30. doi: 10.1186/ar3734 (PMC3392825; doi:10.1186/ar3734)
Supplement: Additional file 1 — Baseline characteristics across participating and non-participating subjects. Table presenting comparison analyses of baseline characteristics across participating and non-participating subjects. [file ar3734-S1.DOC]

**Additional File 1: Baseline characteristics across participating and non-participating subjects**

**Table 1: Baseline characteristics across participating and non-participating subjects**

|  | **Follow-up*** | **Non-follow-up*** | **Comparison** | |
| --- | --- | --- | --- | --- |
| Cohort Characteristics | **(n=58)** | **(n=60)** | **Test Statistic** | **P^$^** |
| Female, no. (%) | 37 (64%) | 41 (68%) | 0.27 | 0.698**^†^** |
| Age, mean ±SD, years | 53 ± 11 | 54 ±14 | -0.34 | 0.736**^‡^** |
| Disease duration, days | 251 (146, 400) | 237 (175, 409) | -0.49 | 0.622 |
| DAS28 | 5.4 (4.6, 6.6) | 5.2 (4.4, 6.3) | 1.07 | 0.287 |
| HAQ | 1.4 (0.9, 2.1) | 1.5 (1.0, 2.1) | -0.76 | 0.445 |
| Larsen Score | 1 (0, 2.5) | 0 (0, 3) | 0.23 | 0.820 |
| ESR, mm/hour | 28 (14, 45) | 24 (11, 40) | 1.135 | 0.256 |
| CRP mg/L | 17 (5, 40) | 8 (4, 24) | 2.14 | 0.032 |
| RF positive, no. (%) | 44 (77%) | 40 (67%) | 1.60 | 0.145**^†^** |
| SJC | 9 (4, 14) | 7 (4, 10) | 1.41 | 0.159 |
| TJC | 15 (6, 18) | 12 (8, 17) | 0.51 | 0.611 |
| CGA on VAS, mm | 49 (20, 60) | 35 (19, 50) | 1.57 | 0.117 |
| PGA on VAS, mm | 40 (26, 58) | 46 (25, 64) | -0.39 | 0.700 |

*Except where indicated otherwise, values are median (25th and 75th percentiles). ^$^Determined by Mann Whitney U Test unless indicated otherwise. ^†^Determined by Fisher’s 2-sided exact test. ^‡^Determined by Student’s t test.
