# Supplementary material for: Baseline serum MMP-3 levels in patients with Rheumatoid Arthritis are still independently predictive of radiographic progression in a longitudinal observational cohort at 8 years follow up
Source: Arthritis Res Ther. 2012 Feb 7;14(1):R30. doi: 10.1186/ar3734 (PMC3392825; doi:10.1186/ar3734)
Supplement: Additional file 2 — Correlation between baseline biomarker measures. Table presenting degree of correlation between novel biomarker measures. [file ar3734-S2.DOC]

**Additional File 2: Correlation between baseline biomarker measures**

**Table 2: Correlation between baseline biomarker measures**

|  | CTX-II | COMP | MMP-3 | TIMP-1 |
| --- | --- | --- | --- | --- |
| CTX-II: rho | 1 |  |  |  |
| n | 54 |  |  |  |
| COMP: rho | 0.56 | 1 |  |  |
| n | 53 | 57 |  |  |
| p-value | 1.22E-05 |  |  |  |
| MMP-3: rho | 0.54 | 0.55 | 1 |  |
| n | 54 | 57 | 58 |  |
| p-value | 3.05E-05 | 9.40E-06 |  |  |
| TIMP-1: rho | 0.33 | 0.50 | 0.42 | 1 |
| n | 54 | 57 | 58 | 58 |
| p-value | 1.55E-02 | 7.97E-05 | 1.14E-03 |  |

Correlation between biomarker measures was assessed by non-parametric Spearman’s rank correlation. Key:
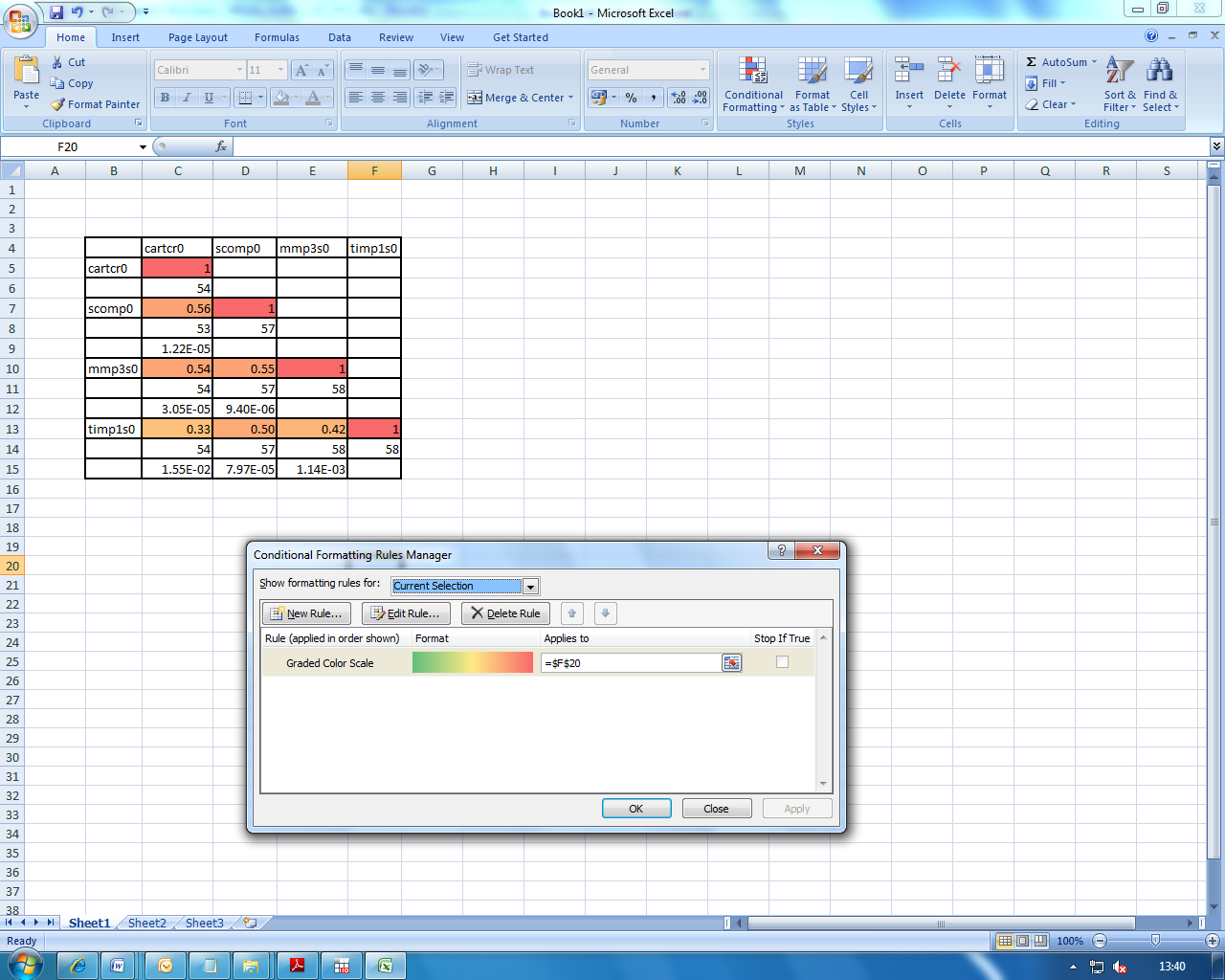


-1 0 +1
